# The diagnosis and management of the Spitz nevus in the pediatric population: a systematic review and meta-analysis protocol

**DOI:** 10.1186/s13643-017-0477-8

**Published:** 2017-04-13

**Authors:** Jean Abboud, Michael Stein, Michele Ramien, Claudia Malic

**Affiliations:** 1 Division of Plastic Surgery, University of Ottawa, 501 Smyth Road, Ottawa, ON K1H 8L6 Canada; 2 Division of Dermatology, University of Ottawa, 501 Smyth Road, Ottawa, ON K1H 8L6 Canada; 3 Paediatric Plastic Surgeon, Children’s Hospital of Eastern Ontario, Ottawa, Canada

**Keywords:** Spitz nevus, Juvenile melanoma, Diagnosis and management

## Abstract

**Background:**

Spitz nevi are uncommon melanocytic neoplasms found in children. Historically, the diagnosis and management of these tumors has lacked consensus among oncologists, pathologists, plastic surgeons, and dermatologists. Once interpreted and treated as a “juvenile melanoma”, many have argued for the benignancy of such tumors in certain patient age groups, encouraging a conservative approach. The lack of consensus surrounding the diagnosis and perceived malignant potential of these tumors has led physicians to approach them on a case-by-case basis and institutional protocols. To date, no evidence-based management guideline exists. The objective of this systematic review is to both collect and appraise the evidence on the diagnosis and management of these tumors.

**Methods:**

A comprehensive electronic literature search will be conducted in PubMed, MEDLINE, Embase, and the Cochrane Library from inception to December 2016. Our search involved collaborating with a healthcare librarian to create a strategy for the OVID/MEDLINE databases. A search of electronic databases for oncology, pathology, plastic surgery, and dermatology abstracts will be performed. Key search terms will include, among several others, “Spitz nevi,” “Spitzoid melanoma,” “juvenile tumor,” and “pediatric”. The language of publication will be restricted to English and French. Wherever data allows, meta-analyses will be used to assess differences between Spitz nevi and the tumor of comparison. Additionally, data extraction and summarization using tables will be performed. This review has been registered with PROSPERO (CRD42016034045).

**Conclusions:**

This review will systematically and comprehensively review diagnostic and management practices associated with the Spitz nevus. This overview of current literature will hopefully provide the foundation for future standardization of clinical practice.

**Systematic review registration:**

PROSPERO CRD42016034045

**Electronic supplementary material:**

The online version of this article (doi:10.1186/s13643-017-0477-8) contains supplementary material, which is available to authorized users.

## Background

Spitz nevi are uncommon melanocytic neoplasms of epithelioid or spindled cells that are found in children [1]. Once treated as a “juvenile melanoma” with malignant potential, these tumors possess a spectrum of clinical and histopathologic presentation that has argued for benignancy [2].

Historically, the diagnosis and management of Spitz nevi has been fraught with confusion [3]. The lack of consensus surrounding the clinical presentation, histopathologic diagnosis, and perceived biologic potential of these tumors has led plastic surgeons, dermatologists, and pathologists alike to approach these tumors on a case-by-case basis. To date, there is no algorithmic approach for either the diagnosis or the management of these tumors [4, 5].

The diagnosis of these tumors is commonly made based on a constellation of clinical and histopathologic features. Spitz nevi are classified as typical or atypical, and some use age as a determinant factor for diagnosis and prognosis. Clinically, Spitz nevi are solitary, well-circumscribed papules/nodules located on the face, upper/lower limb, trunk, or genitalia. The color tends to vary from nonpigmented, red/pink to brown pigmentation. These tumors typically occur in young patients and tend to have a rapid growth phase followed by a static phase. Approximately 50% of cases occur under the age of 10, and approximately 70% are diagnosed in the first two decades of life [6]. They can arise spontaneously or may develop with a preexisting melanocytic nevus. From an epidemiologic standpoint, Spitz nevi are most frequently found in fair-skinned individuals and both sexes are equally affected.

The so-called “atypical Spitz nevus” is a melanocytic proliferation with histopathologic features between a typical Spitz nevus and a Spitzoid melanoma and is associated with an uncertain malignant potential. Few studies have described the dermoscopic features of these tumors [7]. A recent multicenter trial of atypical Spitz nevi documented multicomponent and non-specific dermoscopic patterns and emphasized that these tumors could present in typical spitzoid patterns with amelanotic and hypomelanotic nodules [8].

Pathologic features of Spitz nevi include well-circumscribed borders, spindled or epithelioid cells, giant nevus cells, uniform nuclear and nucleolar enlargement, and maturation of cells deep within the tumor [9]. More concerning features of malignancy potential are present in an atypical Spitz nevus and include numerous and atypical mitoses, marginal mitoses, lack of Kamino bodies, ulceration, lack of maturation, expansile growth pattern, and diffuse p16 negativity [9–11].

There exists a spectrum in both interdisciplinary and intradisciplinary treatment protocols, which range from monitoring, shave biopsy, punch biopsy, and elliptical excision. An elegant study by Metzger et al. in 2013 illustrated contrasting approaches between plastic surgeons and dermatologists [12]. It also demonstrated that dermatologists were managing Spitz nevi in children on a case-by-case basis and were not adhering to their existing guidelines.

The primary objective of this systematic review is to collect and appraise existing literature on Spitz nevi with the intention of informing future diagnostic and management guidelines. Particular emphasis will be placed on the atypical Spitz nevus subgroup.

To ensure scientific rigor, this systematic review protocol was registered with PROSPERO (CRD42016034045), and was adherent with the Preferred Reporting Items for Systematic Reviews and Meta-Analysis Protocols checklist (PRISMA-P), which can be found in an additional file (Additional file 2).

## Methods

### Search strategy

A comprehensive electronic literature search will be conducted in PubMed, MEDLINE, Embase, and the Cochrane Library from inception to December 2016. Our search will involve collaborating with a healthcare librarian to create a strategy for the OVID/MEDLINE databases. We will do a preliminary scan of oncology, pathology, dermatology, and plastic surgery conference abstracts to determine if a full search of the gray literature is necessary. Key search terms will include, among several others, “Spitz nevi,” “Spitzoid melanoma,” “juvenile tumor,” and “pediatric” (see Additional file 1 for full list). The language of publication will be restricted to the English and French language.

### Screening

We will upload the search results and de-duplicate them using the latest EndNote software. Titles and abstracts will go through a level 1 screening by two independent reviewers based on the inclusion criteria, which can be found in full detail in an additional file (Additional file 3). The selected articles will then go through a level 2 screening by both reviewers by reading the full articles.

### Eligibility criteria

Data on patients 18 years and younger with a clinical diagnosis of Spitz nevi will be included in the study. English and French language studies only will be included. Studies must discuss whether nevi were monitored, and/or biopsied, and/or excised. We will evaluate the monitoring protocols, the length of time to follow-up, and the timing of subsequent interventions. We will also document the use of dermoscopy and any other specialized evaluation techniques that do not require excision or biopsy. When the tumors of interest are found to be partially biopsied, we will record where possible the method of biopsy (shave biopsy, punch biopsy, incisional biopsy). When the tumor of interest is removed with the intent of complete removal, we will record where possible the method of removal (shave, punch, excision) as well as the margins and the incidence of recurrence.

### Data extraction and bias assessment

This review will be addressing a number of details surrounding the Spitz nevus; therefore, a broad scope of information will be collected from each study. A list of anticipated items is summarized in Table 1. The PRISMA statement will guide the reporting of our findings. The PRISMA-P checklist is available as an additional file (Additional file 2). The subpopulation will be under 12 years of age and 12 to 18 years old. Gender will be compared to search for a predilection of the disease for male or female. For relevant studies, bias will be assessed using the Cochrane Collaboration Tool for Assessing Risk of Bias [13] and decisions on exclusion or inclusion may rest on the determined level of bias of a study. Systematic reviews will be assessed using A Measurement Tool to Assess Systematic Reviews (AMSTAR) and will be presented in a table [14]. Non-randomized comparative trials will be assessed using Methodological Index for Non-Randomized Studies (MINORS) [15].

**Table 1:** Data collection items

Descriptive details	Population size
	Gender comparison
	Age distribution
	Size of tumor
	Site
	Profile
	History of change
	Family history
	History of excessive sun exposure
Histopathology	Cell type
	Cohesion
	Pigmentation
	Mitosis
	Giant cell
	Epidermal relationship
	Dermal characteristics
	Clark level
	Deep mitosis
	Atypical mitosis
	Nuclear pleomorphism
	High N/C ratio
	Asymmetry
	Solid growth
	Architecture
	Peripheral pagetoidism
	Apoptosis
	Architectural maturation
	Desmoplasia
	Pushing deep border
	Necrosis
	Circumscription
	Prominent nucleoli
	Kamino bodies
Management and outcomes	Excision method
	Margins
	Death
	Sentinel lymph node biopsy and results
	Recurrence rate

### Quality assessment

Each study that is included will be rated based on the Oxford Centre for Evidence-based Medicine (OCEBM) 2011 Levels of Evidence to allow for recommendations to be drawn from the data [16]. Additionally, the quality of evidence regarding reported outcomes will be assessed using the Grading of Recommendations Assessment, Development and Evaluation (GRADE) system principles [17] and a summary table will be produced.

### Statistical analysis

To address the differentiation between Spitz nevi with malignant tumors or tumors with malignant potential, where appropriate, meta-analyses will be carried out by pooling mean differences and odds ratio (OR). If data from the study is not appropriate for meta-analyses, a narrative presentation or summary will be included. Review Manager 5.3 software package will be used for all meta-analyses tables and calculations.

#### Heterogeneity

Due to the complexity of the subject being studied, variability is expected to exist due to factors not limited to measurement error; therefore, a random-effects model (the DerSimonian and Laird method) [18] will be adopted for the meta-analyses with 95% confidence intervals (CI) and significance at the 5% level. We will assess heterogeneity between studies using the chi-squared test [19], and analysis of the inconsistency index (*I* ^2^) will measure how much variation across studies is a result of heterogeneity instead of chance alone (where threshold is I2 > 50%) [20].

#### Publication bias

Where there are sufficient studies included in a forest plot, a funnel plot and test of asymmetry will be used to assess publication bias [21]. Duval and Tweedie trim and fill statistics will be used to adjust for missing studies if publication bias is detected [22–24].

## Discussion

The methodology of this systematic review has been designed to adhere specifically to PRISMA-P protocols in order to be comprehensive and to minimize potential biases. Our objective was to offer a foundation for future diagnosis and management guidelines as comprehensive as possible.

### Limitations

The highly heterogeneous body of literature on Spitz nevi that is likely to be identified during this search may include publications of low quality with small sample sizes. There is also a potential for selection bias and publication bias due to poor indexing of case series studies. The literature surrounding the diagnosis and management of the Spitz nevus is limited. For this reason, our inclusion criteria were very broad. It included publications on Spitz nevi in the adult population if there was some discussion on the pediatric population. Furthermore, texts from oncology, pathology, dermatology, and plastic surgery were similarly evaluated for a multidisciplinary evaluation of existing literature.

## Conclusion

Information from this meta-analysis will be disseminated broadly via presentation at conferences and publication in peer-reviewed journal in order to ensure our findings have a clinical impact on patients. If an inadequate amount of material is generated to perform a quantifiable meta-analysis, we will conduct a narrative review of the literature and discuss observed trends between specialties.

## Additional files

Additional file 1: “Search Strategy” showing the search terms used for each database search engine and the number of results that the search produced. (DOCX 12 kb)

Additional file 2: “PRISMA-P checklist” showing the adherence to the PRISMA protocol checklist. (DOC 84 kb)

Additional file 3: “Inclusion and Exclusion criteria” showing the inclusion and exclusion criteria used when screening the articles. (DOCX 15 kb)

## Abbreviations

AMSTAR: Assessing the Methodological Quality of Systematic Reviews
GRADE: Grading of Recommendations Assessment, Development and Evaluation
MINORS: Methodological Index for Non-Randomized Studies
PRISMA: Preferred Reporting Items for Systematic Reviews and Meta-Analyses
PRISMA-P: Preferred Reporting Items for Systematic review and Meta-Analysis Protocols
