# Supplementary material for: The diagnosis and management of the Spitz nevus in the pediatric population: a systematic review and meta-analysis protocol
Source: Syst Rev. 2017 Apr 13;6:81. doi: 10.1186/s13643-017-0477-8 (PMC5390491; doi:10.1186/s13643-017-0477-8)
Supplement: Supplementary file 1 — “Search Strategy” showing the search terms used for each database search engine and the number of results that the search produced. (DOCX 12 kb) [file 13643_2017_477_MOESM1_ESM.docx]

**Search Strategy - Additional File 1**

PubMed:

(("Nevus, Epithelioid and Spindle Cell"[Mesh]) OR (Spitz Nev* OR Spitz Nevi OR Spitz Nevus OR Juvenile melanoma OR Spitzoid lesion OR Spitzoid tumor))

= 1353 articles

(("Nevus, Epithelioid and Spindle Cell"[Mesh]) OR (Spitz Nev* OR Spitz Nevi OR Spitz Nevus OR Juvenile melanoma OR Spitzoid lesion OR Spitzoid tumor)) **AND** *((children OR youth OR teen* OR pediatric OR pediatrics OR infant* OR newborn))*

= 630 articles

MEDLINE (OVID):

1. "Nevus, Epithelioid and Spindle Cell"/

2. (Spitz Nev* or Juvenile melanoma or Spitzoid lesion* or Spitzoid tumor*).mp. [mp=title, abstract, original title, name of substance word, subject heading word, keyword heading word, protocol supplementary concept word, rare disease supplementary concept word, unique identifier]

= 1045 articles

3. adolescent/ or exp child/ or exp infant/

4. (child or children or youth or adolescent or adolescence or teen* or pediatric* or infant* or newborn* or neonate or neonatal).mp. [mp=title, abstract, original title, name of substance word, subject heading word, keyword heading word, protocol supplementary concept word, rare disease supplementary concept word, unique identifier]

5. 1 or 2

6. 3 or 4

7. 5 and 6

8. limit 7 to (english or french)

= 452 articles (448 duplicates from previous)

EMBASE (OVID):

1. "Nevus, Epithelioid and Spindle Cell"/

2. (Spitz Nev* or Juvenile melanoma or Spitzoid lesion* or Spitzoid tumor*).mp. [mp=title, abstract, heading word, drug trade name, original title, device manufacturer, drug manufacturer, device trade name, keyword]

= 703 articles

3. adolescent/ or exp child/ or exp infant/

4. 1 and 2 and 3

5. limit 4 to (english or french)

= 187 articles (174 duplicates from previous)

The Cochrane Library (OVID):

(Spitz Nev* or Juvenile melanoma or Spitzoid lesion* or Spitzoid tumor*).mp. [mp=ti, ot, ab, tx, kw, ct, sh, hw]

= 3 articles

**Grand total = 650**
