# Supplementary material for: The diagnosis and management of the Spitz nevus in the pediatric population: a systematic review and meta-analysis protocol
Source: Syst Rev. 2017 Apr 13;6:81. doi: 10.1186/s13643-017-0477-8 (PMC5390491; doi:10.1186/s13643-017-0477-8)
Supplement: Supplementary file 3 — “Inclusion and Exclusion criteria” showing the inclusion and exclusion criteria used when screening the articles. (DOCX 15 kb) [file 13643_2017_477_MOESM3_ESM.docx]

Inclusion and Exclusion Criteria - PICO

**Review Question**

To design a protocol for the diagnosis and management of Spitz nevi in the pediatric population.

**Types of study to be included**

We will include diagnostic and outcomes studies that allow for accumulation of management decisions and diagnostic accuracy, including the following

- randomized controlled trials (RCTs) including cluster RCTs
- controlled (non-randomized) clinical trials (CCTs) or cluster trials
- interrupted time series (ITS) studies
- controlled before-after (CBA) studies
- prospective and retrospective comparative cohort studies
- cluster randomized, cluster non-randomized, or CBA studies
- diagnostic case-control studies that separately recruit diseased and non-diseased groups
- systematic reviews on Spitz nevi diagnosis and management protocol
- case series with sufficient study size

We will exclude case reports. We will also exclude studies that focus on correlating years of physician experience with the outcome.

**Participants**

We will include studies examining the pediatric human population (18 years and younger) with lesions suspicious for Spitz nevi or atypical Spitz nevi that make reference or comparisons to the benign Spitz nevus. We will include studies addressing both adults and children if data provided for children are reported separately.

**Interventions**

Of interest are interventions that are conservative versus surgical for the management of individuals with Spitz nevi. We will also consider surgical interventions with variable degrees of margin resection. Conservative interventions include studies that simply observe the lesion by periodical follow-ups and monitor its characteristics. We will also consider interventions where the focus is on histopathological observation.

**Comparator/control**

Comparators will depend on the focus of the study. In studies that look at recurrence rate, the comparator will be those who received a more conservative treatment. In studies that look at age groups that are at higher risk of developing a malignancy or that require a more aggressive management, the comparator will be above 12 years of age. In studies that look at conservative versus surgical management, the comparator will be the conservative option.

**Outcomes**

We will be looking at the following outcomes:

- Accurate diagnosis of Spitz nevus versus a malignancy
- Demographic groups that are more likely to have a malignancy
- Progression to atypical Spitz nevus or malignancy
- Recurrence rate

**Timing**

There will be no restrictions based on timing.

**Setting**

There will be no restrictions by type of setting.

**Language**

We will include articles reported in the English and French languages.
